# Supplementary material for: Biblio-MetReS for user-friendly mining of genes and biological processes in scientific documents
Source: PeerJ. 2014 Feb 27;2:e276. doi: 10.7717/peerj.276 (PMC3940481; doi:10.7717/peerj.276)
Supplement: Supplemental Information 1 [file peerj-02-276-s001.docx]

# Supplementary Materials

These materials contain the following information:

- Section 1: Document search and analysis.
- Section 2: Storage of Information.
- Supplementary Figure 1.
- Supplementary Figure 2.
- Supplementary Table 1.

## Section 1. Document search and analysis

The different information sources accessed by Biblio-MetReS to create a meta-search engine are divided into two groups: Literature Databases (Medline, Pubmed, Plos One, etc) and General Engines (Yahoo, Ask, etc.) (Usié et al., 2011). The organism of interest and the gene(s) and/or biological processes selected by the user are assembled into a query that is launched to each selected information source.

Once the search is completed, Biblio-MetReS eliminates duplicate documents by comparing URLs, Pubmed identifiers, and doi numbers. Once a non-redundant list of documents is assembled, Biblio-MetReS selects the full text of the document to analyze, unless only the abstract is available. It discards all the documents for which the user IP address has no access. Then the tool checks if any of the flagged documents has been previously analyzed and stored in the database, retrieving preprocessed data from that database, if available. At the end of this process, the application analyzes on the fly the documents that were not found in the database, looking for co-occurrence of any gene or protein, and/or for any biological process (if the user included them in the analysis) in sentences, paragraphs, and entire documents. It then stores the relevant analysis of new documents. We note that even though PubMed frequently updates the content of its documents, we assume that these updates will not significantly affect the entities that are found in any given document. Therefore, once analyzed and stored, a document will not be reanalyzed again live unless it is removed first from our database of preprocessed documents.

The method used to find both types of entities (genes/proteins and biological processes/pathways) is exact matching, including all the synonyms for a gene, GO term or pathway term. Gene synonyms are downloaded from NCBI’s central database and GO term synonyms are extracted from the GO category definitions file provided by the GO consortium. The dictionary of genes and their synonyms we use is a merge from those of NCBI and KEGG. The dictionary of biological processes we use is a merge of GO, KEGG and Panther. See the workflow in Supplementary Figure 1 for a summary of the process.

The decision to analyze the co-occurrence in sentences and paragraphs relies on the consideration that, when analyzing full text documents, proximity between the entities implies a more direct relationship between them. If this is not taken into account, a significant amount of noise can be included in the network of possible interactions (Lin, 2009).

## Section 2. Precompiled information in the database.

To save the information related to each document we created a set of seven tables in the database. These tables allow Biblio-MetReS to reduce the execution time and reproduce the same result given by the on-the-fly analysis. The tables are:

1. DocumentsTable
2. SearchTable
3. GenesTable
4. ProcessesTable
5. GenePairsTable
6. ProcessPairsTable
7. GeneProcessPairsTable

Supplementary Figure 2 details the relationship between the tables and their variables. There are some aspects about the variables in each table that have to be clarified. First, a variable in plural refers to a list of elements. In *SearchTable*, the variable *typeProcessSearch* can assume the following values: 0 - the search is done mapping genes and biological processes from GO, Pathways and Panther; 1 - the search is done mapping genes and biological processes from GO; 2- the search is done mapping genes and biological processes from Pathways and Panther; 3- the search is done only mapping genes. In *ProcessesTable* and *GeneProcessPairTable*, the variable *typeProcess* can assume the following values: 0- the biological process is from GO; 1.- the biological process is from Pathway or Panther. Finally, in *ProcessPairsTable* the variable *typeProcessPair* can assume the following values: 0- both biological processes in the pair are from GO; 1- both biological processes in the pair are from Pathway or Panther; 2- one biological process in the pair is from GO and the other is from Pathway or Panther.

## Reference

Lin, J. 2009. *Is searching full text more effective than searching abstracts?* BMC bioinformatics 10:46.

Mao, Y., Van Auken, K., Li, D., Arighi, C., & Lu, Z. 2013. *The Gene Ontology Task at BioCreative IV*. In Proceedings of the Fourth BioCreative Challenge Evaluation Workshop, Vol. 1, pp. 119–127, Bethesda, USA.

Usié, A., Karathia, H., Teixidó, I., Valls, J., Faus, X., Alves, R., & Solsona, F. 2011. *Biblio-MetReS: A bibliometric network reconstruction application and server*. BMC Bioinformatics 12(1):387.

**Supplementary Figure S1**. Algorithmic workflow for the process of network reconstruction in Biblio-MetReS.


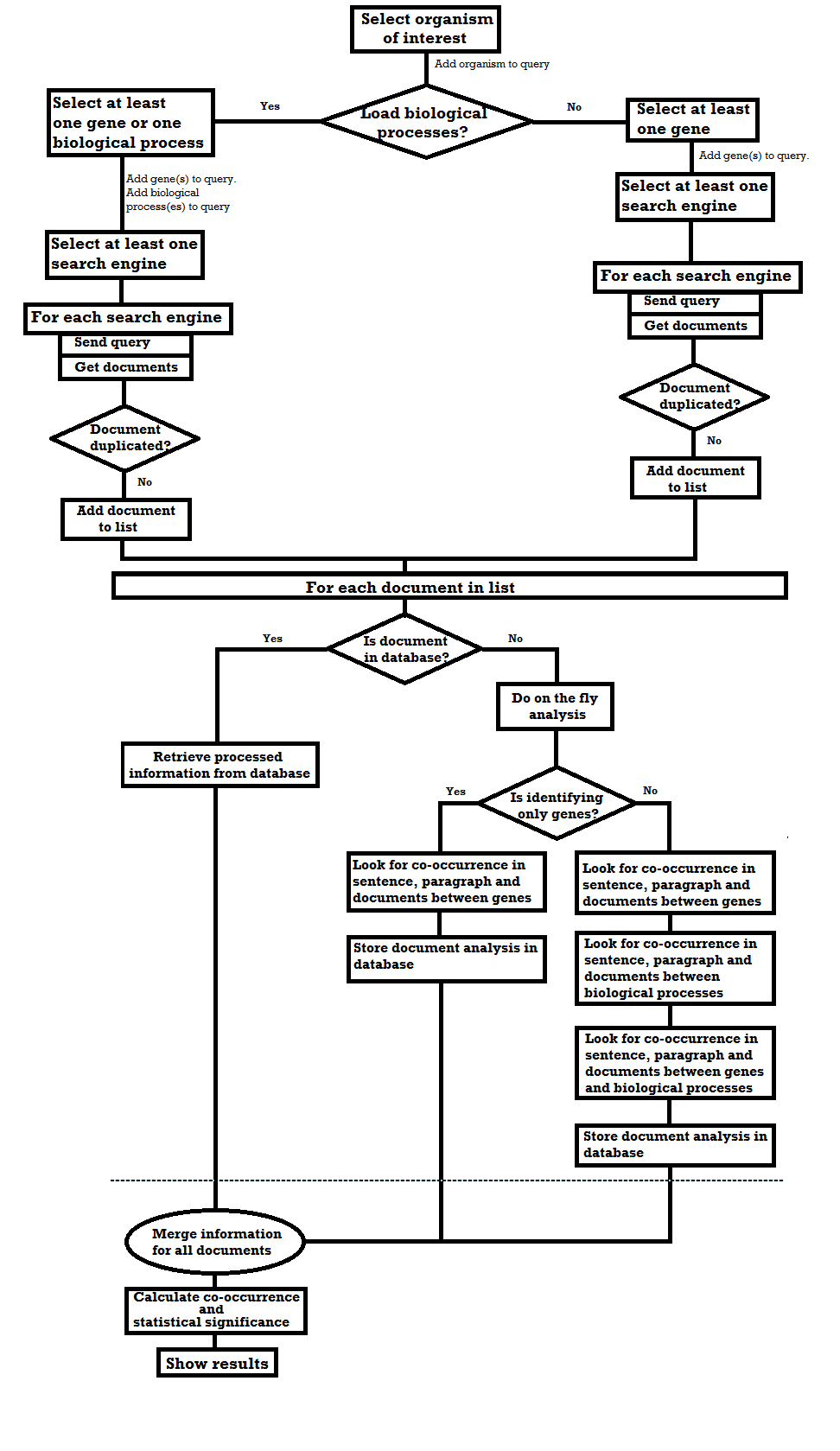


**Supplementary Figure S2.** Database used to store preprocessed information by Biblio-MetReS. Tables and their relationships. See section 3 of these supplementary materials for details.


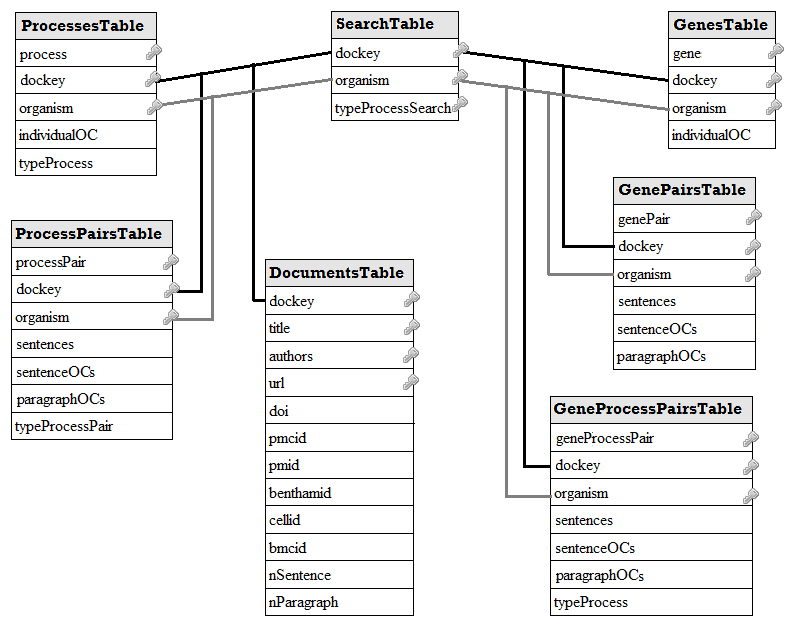


Supplementary Table S1: Organisms and genes used for benchmarking the application.

| **Organisms** | **Pathway** | **Genes to start reconstruction** |  |
| --- | --- | --- | --- |
| *Saccharomyces cerevisiae* | Glycolysis | PGM1; FBA1; CDC19, ***ALL*** |  |
|  | Lysine metabolism | LYS21; ARO8; LYS9, ***ALL*** |  |
|  | RNA processing | MTR3; MPP6; CAF16,; RRP41 ***ALL*** |  |
| *Homo sapiens* | Glycolysis | PGM1; ALDOA; PKLR, ***ALL*** |  |
|  | Lysine metabolism | AADAT; AASDH; AASS ***ALL*** |  |
|  | RNA processing | MTR3; MPP6; CNOT4; RRP41, ***ALL*** |  |
| *Escherichia coli* | Glycolysis | Pgm; fbaB; pykF, ***ALL*** |  |
|  | Lysine metabolism | thrA; dapB; dapF, ***ALL*** |  |
|  | RNA processing | rppH; rhlE; rnr, ***ALL*** |  |
| *Drosophila melanogaster* | Glycolysis | Pgm; Ald; PyK, ***ALL*** |  |
|  | Lysine metabolism | Lkr; CG9547; Gpp, ***ALL*** |  |
|  | RNA processing | Rrp42; Mpp6; Cnot4; Rrp41, ***ALL*** |  |

**Supplementary Table S2**: Number of genes identified by Biblio-MetReS, iHOP, and STRING. Columns labeled MetReS-iHOP, MetReS-STRING, and iHOP-STRING present the number of common genes found by each pair of applications. Columns labeled All presented the number of common genes found by all three applications.

|  |  |  | Glycolysis |  |  |  |  |
| --- | --- | --- | --- | --- | --- | --- | --- |
|  | Biblio-MetReS | iHOP | STRING | MetReS-iHOP | MetReS-STRING | iHOP-STRING | All |
| *E. coli* | 690 | 25 | 569 | 21 | 193 | 19 | 17 |
| *S. cerevisiae* | 620 | 156 | 412 | 60 | 164 | 54 | 38 |
| *D. melanogaster* | 1117 | 50 | 415 | 19 | 73 | 18 | 16 |
| *H. sapiens* | 1163 | 118 | 450 | 44 | 116 | 55 | 35 |
|  |  |  |  |  |  |  |  |
|  |  |  | Lysine metabolism | |  |  |  |
|  | Biblio-MetReS | iHOP | STRING | MetReS-iHOP | MetReS-STRING | iHOP-STRING | All |
| *E. coli* | 307 | 7 | 503 | 6 | 105 | 6 | 6 |
| *S. cerevisiae* | 821 | 15 | 459 | 12 | 212 | 11 | 11 |
| *D. melanogaster* | 899 | 18 | 200 | 10 | 57 | 5 | 5 |
| *H. sapiens* | 1119 | 9 | 161 | 3 | 40 | 5 | 3 |
|  |  |  |  |  |  |  |  |
|  |  |  | RNA | processing |  |  |  |
|  | Biblio-MetReS | iHOP | STRING | MetReS-iHOP | MetReS-STRING | iHOP-STRING | All |
| *E. coli* | 419 | 8 | 486 | 6 | 138 | 7 | 6 |
| *S. cerevisiae* | 502 | 38 | 353 | 26 | 89 | 23 | 20 |
| *D. melanogaster* | 687 | 17 | 104 | 5 | 16 | 3 | 3 |
| *H. sapiens* | 1936 | 54 | 272 | 28 | 87 | 16 | 13 |

**Supplementary Table S3:** Percentage of reduction in Biblio-MetReS run time due to preprocessing in controlled experiments. Pubmed row – results for searching Pubmed exclusively. All databases – results for simultaneous search of all databases available in the application. For each gene and organism, Biblio-MetReS analyzes the documents that are found and stores the analysis. Then, the experiment is repeated and Biblio-MetReS find the same documents but now does not need to reanalyze them, as they have been preprocessed by the previous search. The percentage of time reduction between the first experiment and the second is calculated and the number is presented in the tables. Columns **ALL** represents the results obtained from searching for all the genes together. When all documents found in a new search have been preprocess, repeating this search will not further reduce its run-time, as we can see in some of the entries of the columns **ALL**.

| ***Saccharomyces cerevisiae*** | | | | | | | | | | | | | |
| --- | --- | --- | --- | --- | --- | --- | --- | --- | --- | --- | --- | --- | --- |
|  | **Glycolysis** | | | | **Lysine metabolism** | | | | **RNA processing** | | | | |
|  | **PGM1** | **FBA1** | **CDC19** | **ALL** | **LYS21** | **ARO8** | **LYS9** | **ALL** | **MTR3** | **MPP6** | **CAF16** | **RRP41** | **ALL** |
| **Pubmed** | 97.02 | 99.99 | 97.63 | 0 | 94.80 | 97.54 | 97.55 | 0 | 98.46 | 98.73 | 98.54 | 64.47 | 0 |
| **All databases** | 84.80 | 81.70 | 91.65 | 58.57 | 77.39 | 85.56 | 86.35 | 57.91 | 82.85 | 71.90 | 76.70 | 81.78 | 12.48 |
| ***Homo sapiens*** | | | | | | | | | | | | | |
|  | **Glycolysis** | | | | **Lysine metabolism** | | | | **RNA processing** | | | | |
|  | **PGM1** | **ALDOA** | **PKLR** | **ALL** | **AADAT** | **AASDH** | **AASS** | **ALL** | **MTR3** | **MPP6** | **CNOT4** | **RRP41** | **ALL** |
| **Pubmed** | 99.34 | 98.96 | 99.18 | 0 | 97.73 | 98.64 | 96.69 | 0 | 94.05 | 99.11 | 89.94 | 98.65 | 0 |
| **All databases** | 99.57 | 97.89 | 96.88 | 96.64 | 98.41 | 87.36 | 76.16 | 95.69 | 93.45 | 96.31 | 85.58 | 96.46 | 97.11 |
| ***Drosophila melanogaster*** | | | | | | | | | | | | | |
|  | **Glycolysis** | | | | **Lysine metabolism** | | | | **RNA processing** | | | | |
|  | **PGM** | **ALD** | **PYK** | **ALL** | **LKR** | **CG9547** | **GPP** | **ALL** | **RRP42** | **MPP6** | **CNOT4** | **RRP41** | **ALL** |
| **Pubmed** | 99.30 | 99.41 | 99.10 | 75.69 | 98.89 | 98.99 | 99.34 | 0 | 99.31 | 98.61 | 98.45 | 99.04 | 0 |
| **All databases** | 94.62 | 94.84 | 94.47 | 0 | 80.12 | 12.14 | 94.58 | 0 | 83.27 | 76.18 | 59.47 | 6.23 | 0 |
| ***Escherichia coli*** | | | | | | | | | | | | | |
|  | **Glycolysis** | | | | **Lysine metabolism** | | | | **RNA processing** | | | | |
|  | **PGM** | **FBAB** | **PYKF** | **ALL** | **THRA** | **DAPB** | **DAPF** | **ALL** | **RRPH** | **RHLE** | **RNR** |  | **ALL** |
| **Pubmed** | 97.46 | 97.19 | 91.21 | 0 | 97.93 | 97.37 | 94.95 | 0 | 96.24 | 94.57 | 96.52 |  | 0 |
| **All databases** | 92.69 | 93.00 | 81.94 | 88.63 | 84.76 | 89.65 | 86.31 | 87.15 | 84.42 | 68.82 | 84.97 |  | 85.43 |
